# Pulmonary sarcomatoid carcinoma coexisting with tuberculosis: a case report and literature review

**DOI:** 10.3389/fonc.2024.1492574

**Published:** 2025-01-10

**Authors:** Zhi-Hao Huang, Yu-Fei Zhu, Yun-Yun Zeng, Hui-Yi Huang, Jia-Qi Liu, Wen-Chang Cen, Shan Su

**Affiliations:** ^1^ Department of Oncology, Guangzhou Chest Hospital, Guangzhou, China; ^2^ Graduate School, Guangzhou Medical University, Guangzhou, Guangdong, China

**Keywords:** lung cancer, Tuberculosis, PD-1 inhibitors, case report, pulmonary sarcomatoid carcinoma

## Abstract

Pulmonary sarcomatoid carcinoma (PSC) is a rare non-small-cell lung cancer with sarcomatous components or sarcomatoid differentiation, high degree of malignancy, and insensitivity to chemotherapy or radiotherapy. The management of PSC coexisting with tuberculosis (TB) poses a greater challenge, as it necessitates concurrent administration of both anti-TB and anti-neoplastic therapies. The efficacy of anti-PD-1 immunotherapy in non-small-cell lung cancer is promising, but its safety in patients with co-existent TB remains uncertain. Here, we describe a case of advanced PSC coexisting with TB, which experienced progression-free survival (PFS) of over 36 months after receiving anti-TB and anti-neoplastic therapy composed of chemotherapy, vascular targeting therapy, and PD-1 inhibitors simultaneously. The patient is still being followed up with a satisfactory quality of life. This paper is focused on the characteristics and treatment of PSC and discuss the clinical strategies of lung cancer coexisting with TB.

## Introduction

Pulmonary sarcomatoid carcinoma (PSC) is a rare lung malignancy, which accounts for 0.1%–0.4% of non-small-cell lung cancer (NSCLC) (1). It is poorly differentiated and highly malignant and is insensitive to conventional chemotherapy and radiotherapy. The incidence of lung cancer in patients with tuberculosis (TB) is two to four times higher than that in other populations (2). Patients with concurrent TB and lung cancer usually have a compromised immune system and limited tolerance. The coexistence of these two diseases can significantly impact their overall health, making them more vulnerable to infections and less able to withstand the stress of medical interventions. It is particularly challenging to balance the treatment for TB and cancer, and there is no international guideline and consensus on this. Both lung cancer and TB involve complex changes in the immune microenvironment. In recent years, immunotherapy represented by PD-1 inhibitors has revolutionized the treatment of lung cancer, but the accompanying immune-related side reactions cannot be ignored, which includes the outbreak of TB after the use of PD-1 inhibitors. This paper will show an extremely rare case of PSC complicated with TB who achieved long-term survival after combining chemotherapy, vascular targeting therapy, and immunotherapy.

## Case presentation

**Figure 1:**
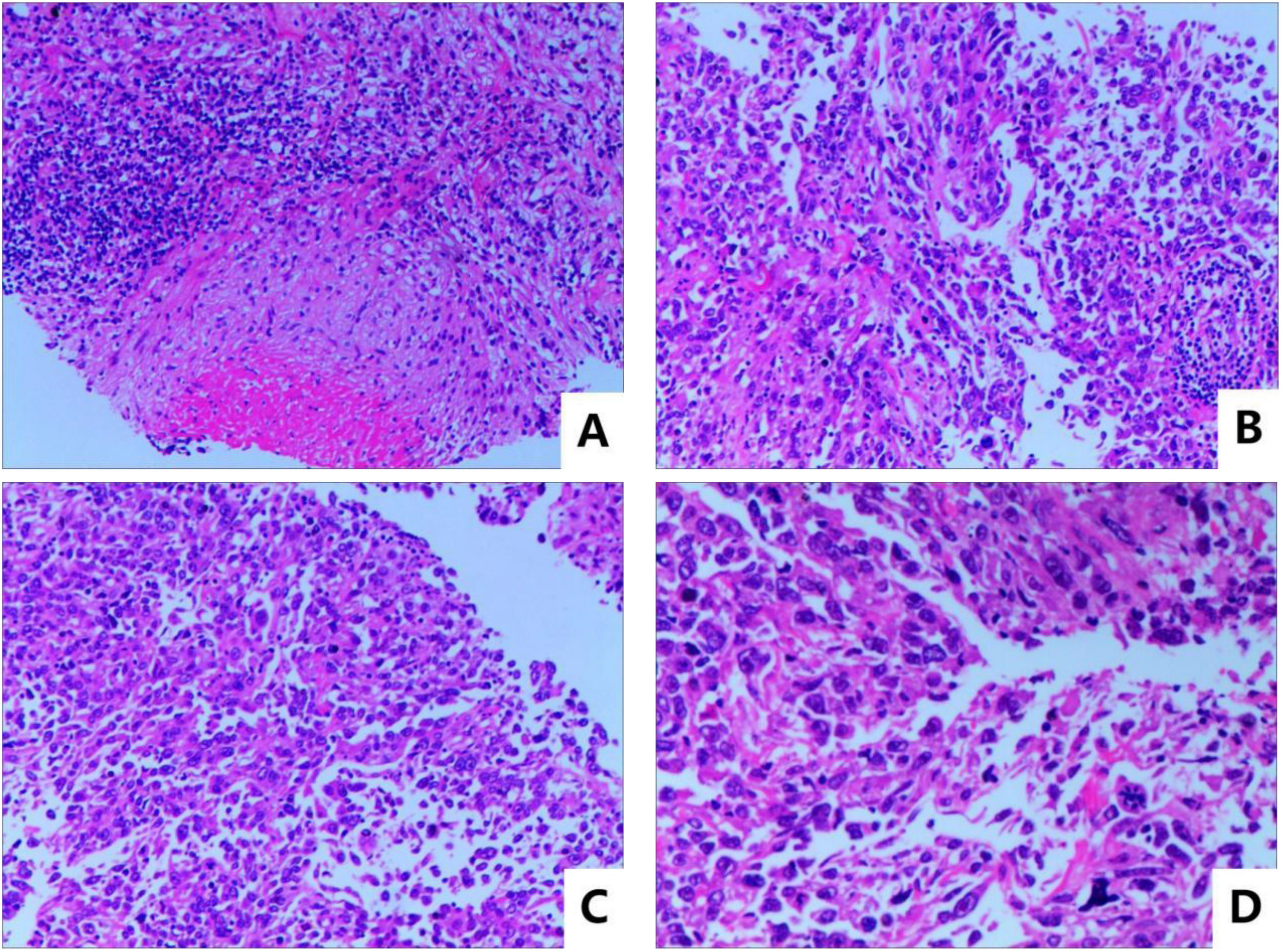
Pathological HE stain image: **(A)** microscopic magnification 100×, **(B, C)** microscopic magnification 200×, and **(D)** microscopic magnification 400×.

**Figure 2:**
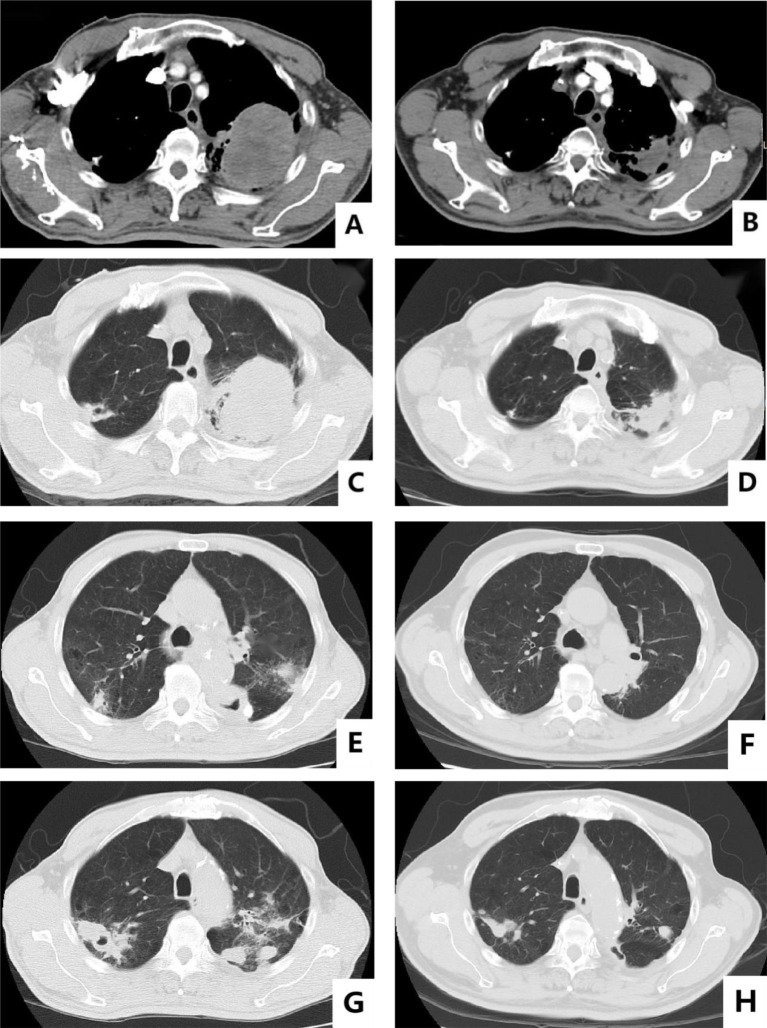
Baseline versus efficacy after four cycles of anti-tumor therapy: **(A, C)** show the images of baseline chest CT mediastinal window and lung window; **(B, D)** show the reexamination images after four cycles of anti-tumor therapy. Changes in pulmonary cavities after anti-TB treatment: **(E, F)** show the changes in the cavity in the left lung before and after treatment; **(G, H)** show the changes in the cavity in the upper right lung before and after treatment.

The patient was a 65-year-old man who claimed to have a history of latent TB infection for more than 30 years and had once received anti-TB treatment for 1 month. He has no history of HIV, and the HIV antibody test is negative. He had a history of type 2 diabetes mellitus for 3 years, and his blood glucose was well controlled with metformin and gliclazide. He also has a smoking history of over 40 years and still has not been able to quit smoking. The patient presented with cough, expectoration, and progressive weight loss. Chest computed tomography (CT) examination showed secondary pulmonary TB in both lungs with cavities in the right upper and left lungs. The patient’s sputum test was positive for acid-fast bacilli, sputum TB-RNA, and rapid mycobacterium culture, which was identified as *Mycobacterium tuberculosis*, and the drug sensitivity test indicated that isoniazid, rifampicin, pyrazinamide, and ethambutol were all sensitive. After anti-TB treatment with isoniazid, rifampicin, pyrazinamide, and ethambutol, the patient’s cough and expectoration symptoms were significantly improved, but intermittent hemoptysis occurred. Three months later, the patient began to feel a dull pain in the left anterior chest, and the chest CT showed a new mass in the left upper lung. Sputum smear was rechecked for acid-fast bacilli, and nucleic acid test was negative. Positron emission tomography computed tomography (PET/CT) scan showed lesions in the left upper lung, pleura, and ribs. CT-guided fine needle aspiration biopsy revealed that the tumor cells were oval or flat spindle shaped; some of them had prominent red nucleoli, moderate amount of cytoplasm, and diffusely arranged in sheets; and mitotic figures were easily seen. Epithelioid cell mass aggregation, necrosis, and lymphocyte infiltration were observed focally (**Figure 1**). Immunohistochemical revealed the following: CK focal (+++), Vimentin diffuse (+++), CD99 (−), LCA (−), Ki67 hot area approximately 65% (+), S-100 (−), Desmin (−), SMA (−), AFP (+), Bcl-2 focal (+), CD3 (−), CD20 (−), and CD68 (+). The diagnosis was sarcomatoid carcinoma, and the clinical tumor stage was cT4N2M1c IVB (UICC/AJCC 8th version). The patient did not undergo further genetic testing and PD-L1 testing due to financial difficulties. Considering that the patient’s sputum smear has turned negative after intensive anti-TB treatment, we administered first-line antineoplastic treatment with paclitaxel (175 mg/m^2^), carboplatin (AUC5), and endostar (210 mg) + sintilimab (200 mg), while simultaneously giving isoniazid and rifampin as consolidation anti-TB treatment. The patient developed grade III myelosuppression after four cycles of antineoplastic therapy, which improved after treatment with recombinant human granulocyte colony-stimulating factor, and CT showed that the mass in the left upper lung was significantly reduced (**Figure 2**), with the treatment efficacy evaluated as partial response (PR) (Response Evaluation Criteria in Solid Tumors, RECIST v1.1). Subsequently, the patient began to receive maintenance treatment with sintilimab 200 mg + Endostar 210 mg every 3 weeks, and the anti-TB drugs were discontinued after 1 year of treatment. A re-examination of chest CT after the end of anti-TB treatment indicated that the bilateral pulmonary cavities had disappeared (**Figure 2**). There were no liver function abnormalities and myelosuppression during this period, and regular efficacy evaluation showed stable disease (SD) (RECIST v1.1). At the 22nd month of anti-tumor treatment, the patient experienced lumbar pain. Magnetic resonance imaging (MRI) revealed nodular abnormal enhancement lesions in the second and third lumbar vertebrae, which were regarded as metastatic lesions. Subsequently, palliative radiotherapy of the lumbar vertebra was administered at a dose of 30 Gray (Gy)/10 Fraction (F). At present, the patient has received maintenance treatment for more than 30 months, and the chest lesions are still stable.

## Discussion

The 2023 WHO Global Tuberculosis Report shows that in 2022, the estimated number of new TB cases in China was 748,000, accounting for 7.1% of the total estimated new cases globally (10.6 million). China is the third highest TB-burdened country in the world, only after India and Indonesia, and the corresponding estimated incidence rate is approximately 52 per 100,000 (3). Studies have shown that repeated inflammatory stimulation caused by chronic *M. tuberculosis* infection is one of the causes of malignant tumors, suggesting a causal link between pulmonary TB and lung cancer (4). On the other hand, the low immune function of patients with malignant tumors and the nutritional deficiency, infection, and psychological factors caused by the disease may further weaken the body’s resistance to *M. tuberculosis* infection (5). In this case, the patient suffered from TB more than 30 years ago and stopped the drug after 1 month of anti-TB treatment. He was in a state of chronic TB infection for a long time, which may be related to the occurrence of tumors.

PSC is a rare non-small-cell lung carcinoma (NSCLC) with sarcomatous component or sarcomatoid differentiation, which is characterized by high invasiveness, early metastasis, and poor prognosis (6). In 2004, the World Health Organization (WHO) classified PSC into five subtypes: pleomorphic carcinoma, spindle cell carcinoma, giant cell carcinoma, carcinosarcoma, and pulmonary blastoma (1). It continued to be used in the 2015 version of lung tumor classification. PSC mainly occurs in elderly men aged 65–75 years old with a common history of heavy smoking (7). It contains central and peripheral types, and the clinical manifestations lack of specificity. The common symptoms of NSCLC such as cough, expectoration, chest tightness, and hemoptysis can occur in PSC, which are mostly related to the invasion range and size.

Surgery is still the main treatment for early PSC, and traditional radiotherapy and chemotherapy are important treatments for unresectable PSC. However, both the postoperative recurrence time and the efficacy of chemoradiotherapy are not ideal. According to the report, the median recurrence time of postoperative PSC patients was only 11.3 months, which was significantly lower than the 61.4 months of other NSCLC patients (p=0.0001), and the 5-year survival rates were 24.5% vs. 46.3%, respectively (p=0.01) (8). In another retrospective study of 97 patients with advanced PSC who received at least two cycles of chemotherapy, the disease progression rate reached 69%, and the median progression-free survival (PFS) and overall survival (OS) were 2 months (CI 95%, 1.8–2.3) and 6.3 months (CI 95%, 4.7–7.8) (9).

PSC is considered to be a kind of NSCLC with high mutation probability. Studies have shown that approximately 69%–80% of PSC patients contain at least one gene mutation, but patients with common sensitive mutations also seem to benefit less from targeted therapy than other NSCLC (10, 11). The epidermal growth factor receptor (EGFR) mutation rate is controversial in PSC patients, from approximately 0% to 28% (12). At present, there is still a lack of large-sample prospective clinical trials on the efficacy of EGFR-tyrosine kinase inhibitors (EGFR-TKI) in the treatment of PSC, and only a few small-sample case reports and retrospective observations are available; the results were quite different (13, 14). A study showed that the rate of anaplastic lymphoma kinase (ALK) mutation in 141 PSC patients was 3.5% (15). In another cohort study from China, the detection rate of ALK in 82 PSC patients was 10.7%, the objective response rate (ORR) after crizotinib treatment was 89%, and the 5-year OS rate was 88.9% (16). In other rare mutations site in PSC, such as mesenchymal–epithelial transition (MET), Kirsten rats arcomaviral oncogene homolog (KRAS) have also been reported, and the corresponding targeted drugs have shown certain efficacy, which needs to be further confirmed (17–19).

Based on KEYNOTE-189 and KEYNOTE-042, the treatment mode of immune checkpoint inhibitors (ICIs) monotherapy or combination chemotherapy according on the expression of programmed death receptor ligand-1 (PD-L1) is also instructive for the treatment of advanced PSC. Studies have shown that the positive rate of PD-L1 in PSC is significantly higher than that in other NSCLC patients, reaching more than 50%, suggesting that PSC may be effective for ICIs (20). There is a case report that programmed cell death-1 (PD-1) inhibitor treatment of PSC patients with brain metastases achieved partial response (PR), and the brain metastases completely disappeared (21). Another PSC patient with liver and small intestine metastasis was converted to immunotherapy after the progression of chemoradiotherapy, the abdominal lesion was relieved, and the overall survival time was more than 5 years (22). Anti-angiogenesis is an important method in the treatment of NSCLC, and it is also applicable to PSC, which is characterized by significant vascular invasion. Oizumi et al. have compared the efficacy of chemotherapy combined with bevacizumab and chemotherapy alone in the treatment of PSC. The median PFS of the combination group was 4.2 months, which was significantly higher than 1.2 months of the chemotherapy alone group, and the median OS was 11.2 months vs. 7.9 months, respectively (23). Another small sample clinical study evaluated the efficacy of endostar combined with chemotherapy in the treatment of PSC, which also had advantages over chemotherapy alone (24).

The treatment of cancer coexisting with TB is a complex process: on the one hand, blood toxicity and liver toxicity associated with anti-TB drugs overlap with the adverse reactions of cytotoxic chemotherapy drugs, and the optimal time of drug administration has not been determined; on the other hand, PD-1 blockade has been observed to exacerbate *M. tuberculosis* infection in animal experiments (25), and it is difficult to predict whether ICIs will also cause TB infection outbreaks in patients with lung cancer complicated with TB. Theoretically, chemotherapy and ICIs do not increase the risk of TB in cancer patients, but the latter may lead to reactivation of latent infection, and long-term steroid use is associated with an increased risk of TB. A retrospective study analyzed the safety and efficacy of concurrent chemotherapy and anti-TB therapy in 33 patients with lung cancer coexisting with TB and 66 patients with lung cancer alone as comparison. After two cycles of chemotherapy, the ORR was 33.3% vs. 42.4% (p=0.383), the time to treatment failure (TTF) was 5.6 months vs. 7.0 months (p=0.175), and the median OS was 14.0 months vs. 17.0 months, respectively (p=0.312), which showed no statistical difference. The main grade 3 adverse effect was hematologic toxicity, and the incidence of liver damage was similar in the two groups (27% vs. 26%). After 3 months of anti-TB treatment, all patients in the lung cancer coexisting with TB group achieved a radiographic improvement, sputum acid-fast bacilli smear turned negative, and no TB recurrence was observed (26). Another cohort study examined the efficacy of EGFR-TKI in patients with lung cancer coexisting with TB. Compared with patients with lung cancer alone, the median PFS of the two groups was 7.47 months vs. 11.77 months (p=0.038), and the median OS was 13.0 months vs. 20.0 months (p=0.017), respectively. It concluded that lung cancer coexisting with TB patients had a poor response to EGFR-TKI treatment (27). A cohort study from the team of Guangzhou Chest Hospital included 98 lung cancer patients, in which 45 had coexisting active TB, 21 had coexisting latent TB, and 32 had coexisting obsolete TB. There was no significant difference in efficacy between the three groups after receiving antitumor therapy including PD-1 inhibitors, two cases of TB recurrence in the active TB group, and no TB recurrence in the other two groups. The incidence of hematologic toxicity above grade 3 was 11.1%, and the overall tolerance was good (28).

## Conclusion

In this paper, we report an extremely rare case of PSC coexisting with pulmonary TB. After receiving standard full-course anti-TB therapy combined anti-tumor therapy with chemotherapy, vascular targeting, and PD-1 inhibitor, the patient achieved ideal efficacy with mild adverse reactions, which is worthy of clinical reference. It should be noted that the genetic status and PD-L1 expression level of this patient are unknown, and large prospective clinical studies in the field of cancer coexisting with TB are currently scarce, which still need to be further explored.

## Data Availability

The original contributions presented in the study are included in the article/**Supplementary Material**. Further inquiries can be directed to the corresponding author.
